# Placing Human Behavior at the Center of the Fight to Eradicate Polio: Lessons Learned and Their Application to Other Life-Saving Interventions

**DOI:** 10.1093/infdis/jiw546

**Published:** 2017-06-30

**Authors:** Sherine Guirguis, Rafael Obregon, Michael Coleman, Benjamin Hickler, Gillian SteelFisher

**Affiliations:** 1 Health Section and; 2 Communication for Development, United Nations International Children's Emergency Fund, New York, New York; and; 3 Harvard Opinion Research Program, Harvard T. H. Chan School of Public Health, Boston, Massachusetts

**Keywords:** Human behavior, polio eradication, social and behavioral change, communication for development, social data, GPEI.

## Abstract

Today, acceptance of oral polio vaccine is the highest ever. Reaching this level of acceptance has depended on decades of engaging with communities, building trust amid extraordinary social contexts, and responding to the complex variables that trigger behavioral and social change. Drawing on both the successes and setbacks in the 28 years of the Global Polio Eradication Initiative (GPEI), this article articulates what happened when the GPEI began to pay more attention to the dynamics of human and social behavior change. Three particular lessons for other health and immunization programs can be drawn from the experience of GPEI: change begins from within (ie, success needs institutional recognition of the importance of human behavior), good data are not enough for good decision-making, and health workers are important agents of behavior change. These lessons should be harnessed and put into practice to build demand and trust for the last stages of polio eradication, as well as for other life-saving health interventions.

In 1988, the World Health Assembly (WHA) endorsed the ambitious goal of eradicating a vaccine-preventable disease—polio—for only the second time in history. After the protracted but successful eradication of smallpox, enthusiasm from the global health community to embark on another global eradication initiative was mixed. In many forums, skeptics and champions debated the merits and risks of global polio eradication. The epidemiology and biology of the disease, the efficacy of oral polio vaccine (OPV) versus that of inactivated (injectable) polio vaccine (IPV), the political commitment required, the cost-benefit analysis to the world, and the risks of a vertical eradication program undermining immunization programs were all at the forefront of deliberations [1, 2].

Twenty-eight years have passed since the goal was adopted by the World Health Organization, the United Nations Children’s Emergency Fund (UNICEF), Rotary International, and the Centers for Disease Control and Prevention. None anticipated it would take this long to achieve, and polio has still not yet been eradicated. But the world is closer than it has ever been. The issues that were heavily debated in the lead up to the 1988 WHA declaration have continued to pose challenges and generate innovative solutions throughout the long journey to eradication. But one critical issue, minimized in those deliberations and subsequently often overlooked in the design and implementation of eradication programs, has arguably been the lynchpin to success or failure in nearly every country that has eliminated the virus: human and social behavior.

As the initiative accumulated success and setbacks, it became evident that the success of eradication depends not only on technical innovation or organizational and political commitment, but also on successfully engaging communities for prolonged periods. In parallel, the setbacks the global eradication program has faced in different settings have all too often arisen because individual and community engagement were inadequate or not prioritized.

For the field of public health, the lesson to place human factors—how people think, make choices, and behave—at the center of disease control initiatives is a lesson that the world cannot afford to overlook again. Immunization programs, health programs, and, more broadly, development programs can benefit from an approach based on a more deliberate and strategic consideration of human factors.

There is no vaccine against resistance or refusals that are rooted in social, cultural, religious and political contexts. No supply chain can overcome issues of gender-based decision-making in households. Medical approaches alone cannot address certain community concerns…. These challenges demand effective communication action… [3].

Today, polio survives in limited reservoirs in only 3 countries: Nigeria, Afghanistan, and Pakistan. Community acceptance of OPV, even in these countries, is the highest it has ever been: 99% of parents in the remaining polio reservoirs accept OPV for their children each time it is offered [4]. Decades-long engaging with communities, building trust, and responding to the complex determinants of behavioral and social change has brought the world closer than ever to eradication. The experience has built an exceptional wealth of lessons in the realms of vaccine demand, behavior change, and organizational culture and management.

This article articulates what happened when the Global Polio Eradication Initiative (GPEI) began to pay more attention to the dynamics of human and social behavior change and to the lessons that can be drawn for other health and immunization programs.

This analysis draws on key reports and documents of polio eradication efforts (eg, peer-reviewed literature and reports by the GPEI and the GPEI Independent Monitoring Board), with a particular focus on social, behavioral, and communication dynamics. The article is heavily informed by the authors’ long-time engagement with policy, evidence and research, and implementation aspects of the global polio eradication efforts, which include, among many others, the following: direct involvement of 4 coauthors in field-based and technical assistance to polio eradication efforts, leadership by or involvement of 3 coauthors in systematic data collection to inform communication and social mobilization strategies, and regular participation by 2 coauthors in polio reviews and capacity development efforts at the country level. The article focuses primarily on lessons learned, because social, behavioral, and operational dynamics of polio eradication have been widely documented in the peer-reviewed literature and by reports from the GPEI Independent Monitoring Board and others.

## Lesson 1: Change Begins From Within—Success Needs Institutional Recognition of the Importance of Human Behavior

Perhaps the most critical lesson that has emerged is that behavior change must come first from individuals and organizations themselves before change can be seen in the communities with whom we seek to engage. This lesson is exemplified by the experience of the GPEI in India, beginning in the early 2000s.

In 2002, 1600 children were paralyzed by polio in India, a rise from 268 the year before, which meant India housed 63% of the world’s polio cases [5]. In pockets in Uttar Pradesh and Bihar, which are Muslim states and host the country’s 2 final reservoirs of transmission, vaccination teams were often met by angry families. Amid deplorable environmental and health conditions, underserved families were unable to believe that a government program was offering universal free vaccination for a single disease and that this vaccine was intended to help and not harm their children. In these communities, households would band together, putting up block resistance to vaccination and thereby allowing the virus continued refuge to thrive. The India program was in dire need of new solutions.

In 2001, in Uttar Pradesh, an innovative network of women (the Social Mobilization Network [SMNet]) was built by UNICEF to serve as a bridge between the polio program and the communities it served by facilitating dialogue and trust in communities with the largest burden of poliovirus. Mothers from the community were trained as community mobilization coordinators and tasked with speaking to other mothers about children’s health, the importance of taking the polio vaccine repeatedly, and quelling concerns about its safety.

The SMNet initially exacerbated community tensions because the idea of women working outside the household was yet another unwelcome intrusion on the religious beliefs of many in these underserved Muslim communities. With time, the female network adapted and began to show progress. Women worked in pairs, and older women were recruited to enhance moral and social acceptability. The numbers of vaccine refusals and unvaccinated children declined in areas with community mobilization coordinators, and communities were beginning to participate in—and even lead—polio eradication activities. In Uttar Pradesh, the network began to demonstrate its value as an integral part of India’s polio eradication strategy. The SMNet expanded to Bihar in 2005, with appropriate contextual adaptations that relied more heavily on government community-health workers. When India saw its last polio case, in 2011, the SMNet was widely recognized as one of the pivotal strategies of India’s success. Today, SMNet is on the leading edge of transition as it focuses more on health issues beyond polio and state governments cover more of the operating costs [6].

The India experience demonstrated several new lessons for the GPEI: (1) community demand and support could either facilitate or erode progress toward eradication, (2) women engaging with other women is a successful strategy to build trust and facilitate access to more children [6], and (3) innovations need time and space to fail and adapt, to benefit from local knowledge, and to be monitored to see whether they can breed success.

The GPEI and the individual organizations composing it have responded to each of these lessons with varied conviction over the subsequent years. Although the behaviors and commitment of parents and communities were accepted in principle as an important component of program design, they remained on the fringes of operations in most countries and in global policy—an element that would be nice to have if resources allowed. Polio eradication was still driven by epidemiologists and their data, and this remains the core strategy of eradication today.

As a result, communication and social mobilization remained drastically underresourced until 2010, when the global program again faced a crossroads and the Bill and Melinda Gates Foundation made a large contribution to UNICEF—the lead agency for communication and social mobilization—to deliver better performance in this area.

As the importance of engaging with communities was finally beginning to translate into action, communication staffing structures were built at national and subnational levels, and dedicated social mobilization networks were established in Nigeria, Pakistan, and Afghanistan. Yet these networks were assembled incrementally, taking years to get to the required scale, and—in Pakistan and Afghanistan—were staffed by an overwhelmingly male workforce.

Despite the lessons from India and the vast amount of literature supporting female-to-female peer engagement, employment of women to build trust with other mothers for polio in the most conservative areas was only starting to be accepted by many governments and development officials in the GPEI by 2015. Nigeria scaled up much more quickly than Afghanistan and Pakistan and staffed its network (the volunteer community mobilizers) with 99% women in the high-risk conservative Muslim northern states. An earlier decision in Pakistan and Afghanistan could have contributed to much faster implementation of and more-effective vaccination strategies in Pakistan and Afghanistan.

The World Bank’s 2015 World Development Report posited that “development professionals, like everyone else, are themselves subject to the biases and mistakes that can arise from thinking automatically, thinking socially, and using mental models.” To demonstrate this, the 2015 World Development Report team studied how World Bank staff members interpreted data. When World Bank staff were asked to interpret data about something ideologically and politically neutral—skin cream—versus data that was more politically charged and related to their core area of work—poverty reduction—the survey found that, in their core area of work, staff were more likely to interpret new data in a manner consistent with their prior views.

Development professionals can be susceptible to a host of cognitive biases, can be influenced by their social tendencies and social environments, and can use deeply ingrained mindsets when making choices [7].

Such implicit biases may partly explain why it took the GPEI almost 2 decades to recognize the importance of human and social factors in disease control and a decade to fully implement a proven and effective strategy to build trust in just 3 polio-endemic countries. Such biases may also explain why it took several additional years to recruit predominantly female health and social mobilization workers in areas that—because of cultural or security concerns—the program assumed would not welcome female health workers.

Concerns about using a female workforce may be valid in some areas, but they have been overcome in most areas of Pakistan, suggesting that use of such a workforce is possible even in very conservative communities [8]. By contrast, in Afghanistan, the sex-related lesson remains unheeded. The given rationale is that engaging female workers in southern and eastern Afghanistan is socially unacceptable and nearly impossible to implement. As a result, the Immunization Communication Network—Afghanistan’s social mobilization network—is only 12% female, despite 62% of caregivers in low-performing districts stating they feel it would be “most acceptable” in their community to have at least 1 female among health workers going door to door [9].

Organizations need to be more aware of these biases, and organizations should implement procedures to mitigate them…. Instead of penalizing failure or burying findings of failure, organizations need to recognize that the real failures are policy interventions in which learning from experience does not happen [7].

## Lesson 2: Good Data Are Not Enough for Good Decision-Making

A second reason that human behavior may have been overlooked in program design was that, until 2011, there was a dearth of credible social data that demonstrated behavioral and social insights in any country. To mitigate enduring biases in the polio program that considered social and behavior factors to be optional strategies of disease control and to remove the subjectivity of recommended and implemented behavioral interventions, UNICEF undertook an initiative to produce credible social data that could be used to inform global policy and behavior change strategies across polio-affected countries. An innovative partnership between UNICEF and the Harvard Opinion Research Program at the Harvard T. H. Chan School of Public Health was established in 2013 to conduct quantitative knowledge, attitudes, and practice polls.

Knowledge, attitudes, and practice studies had been used by the program for many years. The innovation lay in (1) producing high-quality data, using rigorous survey methods in countries with low research infrastructure, which made it more difficult for the program to dismiss results as unreliable or methodologically flawed; (2) developing a system of standardized metrics that could be used to detect commonalities and differences among multiple polio-affected countries and still provide countries with the flexibility to measure issues specific to each context; (3) collecting representative data at lower geographic levels, which allowed the program to develop tailored behavioral strategies in local high-risk areas; and (4) prepositioning a set of indicators and questions that were cleared in advance by the partnership and countries so that polls could be rapidly conducted when needed. Finally, the polls used a multidisciplinary model of health behavior and trust that draws on political science and emergency response paradigms to measure 2 things never before been quantified in the polio context: community trust in the polio program and demand for other services beyond OPV. It was hoped that these insights might identify new strategies for reaching chronically missed children that the program was unable to reach through other approaches.

Together with Harvard researchers, a conceptual framework was developed that measured 4 dimensions of trust: competence, morality, compassion, and concern for the child. These dimensions were assessed at 3 levels: the vaccine, the health workers who visited families, and the institutions perceived as delivering polio services. In addition, the polls asked parents and other caregivers what services were a priority for their families, as a means to assess community demand for services beyond OPV. This was another component that was often assumed by the program, which nearly automatically offered standard commodities, such as zinc or oral rehydration salts, to supplement OPV in areas where demand for other services was hindering vaccine uptake.

The data demonstrated striking and consistent patterns across the remaining polio-endemic countries: in the reservoirs with insecurity and the heaviest burden of virus transmission, community trust in most metrics—OPV, health workers, and the program—was lowest. Trust in the health worker was the most marked: for example, at the height of Taliban-led inaccessibility in the Federally Administered Tribal Areas of Pakistan (2014), only 24% of people living there trusted recent vaccinators “a great deal,” compared with 61% in other areas. In Borno State of Nigeria, the stronghold of conflict between Boko Haram and government forces, only 48% of people trusted their vaccinators “a great deal,” compared with 70% in the other high-risk areas [10].

In addition, the data showed that, although individuals’ intent to vaccinate repeatedly was often very high, the perceived social norms supporting this intent (eg, the fraction of caregivers who felt that their neighbors also vaccinate their own children or the fraction who said the child’s grandparents, community leaders, or neighbors thought that giving polio drops was a good idea) in all high-risk areas were not. These are worrying indicators for the maintenance of polio-free status, as it suggests that people accept the vaccine reflexively today but that this acceptance is fragile and could be threatened without stronger support at community, organizational, and policy levels. This could be a particular vulnerability for sustaining progress as the disease is eliminated and people’s risk perception of it dwindles [11].

Before the August 2016 polio outbreak in Borno that dragged Nigeria back to polio-endemic status, 2015 polling data showed that caregivers’ belief in negative rumors about OPV was 19% in research-accessible local government areas of Borno as compared to 4% in the other high-risk areas. Only 43% of caregivers in these parts of Borno said that they intend to vaccinate their children “every time” OPV is offered, compared with 67% in other high-risk areas [12]. This data should have been a warning signal to program leaders that uptake of vaccine in Borno could be substantially jeopardized even when households were reached by health workers. Particularly in an area with insecurity and intermittent or no access to the program, the task of ensuring acceptance and demand among all whom the program had access to could have been more aggressively acted upon before wild poliovirus was detected once again.

A set of standardized, credible, and regularly collected data has been of enormous benefit to the polio program: it has shifted behavioral strategies at global and country levels (see lesson 3), improved the integration of behavioral and social considerations into the operations of the program, and demonstrated the value that communications and social mobilization could have to reach more children with OPV. Yet it has also shone a spotlight on substantial vulnerabilities in the program that should not be disregarded.

Social data have been used only when there have been individuals and organizations with the skills and commitment to raise its significance to a level on par with that of epidemiological data and to mobilize stakeholders to act upon it. It has been more likely to be ignored when the evidence goes against popular, long-held beliefs of program practitioners or leaders or when it points to risks that the epidemiological data have not yet identified. Where it could be dismissed or misinterpreted by those that have a vested interest in the status quo, it has been too easy to do so.

## Lesson 3: Health Workers Are Important Agents of Behavior Change

Where social data generated through polls and other sources have been used, they have led to critical shifts in how the polio program communicates and engages with communities. Polling data demonstrated that there was a challenge of health worker trust in and negative social norms about polio vaccination among segments of the population that were most critical for eradication. These data led to a new global communications strategy, in 2015, that focused on (1) promoting all vaccination as a social norm, (2) creating mass media campaigns that were more emotionally resonant and less exclusively driven by information and awareness, (3) building trust and goodwill for health workers by humanizing them, and (4) building a new brand for the polio program that could reinforce positive reforms recently implemented.

Perhaps the most substantial shift of the new strategy was a focus on health workers as agents of behavior change. As Ashraf has noted, “Health isn’t something that can be handed to people; it is a state that they must produce themselves by interacting with a health care system…providers and recipients co-create health” [13].

**Figure 1.**
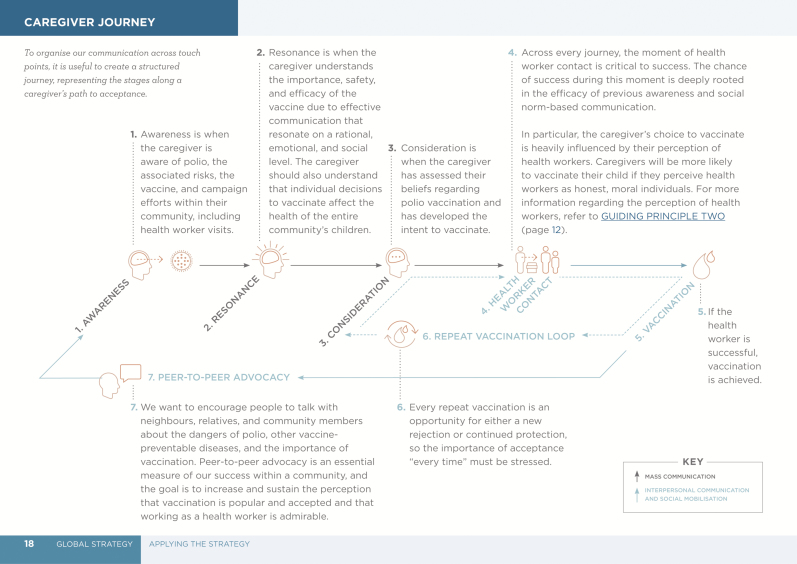
The caregiver journey to OPV acceptance. The figure originally appeared in the Polio Communication Global Guide [14p19].

Unlike smallpox eradication, polio requires each child to be vaccinated multiple times, with children in some contexts receiving ≥50 vaccinations by the time they 5 years old. Repeated vaccination relies on a positive interaction between health worker and caregiver every single time. Knowledge, competence, compassion for children, and morality—the 4 metrics in the model of trust—must be demonstrated at every caregiver–health worker interaction and supported by all communication strategies before and after (Figure 1) [14].

Focusing on health workers as agents of behavior change meant that the communication program was no longer relying exclusively on social mobilization networks to build trust between the program and communities. Introducing social components into the operational tactics of the program was a significant step forward in integrating human and social principles into core eradication strategies. Communication based on community insights and behavioral science was no longer an adjunct to the program but a central pillar of it.

The strategy was first rolled out in Pakistan in 2015. Vaccinators would be exposed to a new, integrated approach that incorporated interpersonal communications throughout the full vaccinator training curriculum. Previously, interpersonal communication skills were covered in a short standalone module that was poorly delivered if at all. Additionally, motivating health workers by meeting their needs for information (how to get paid, how to ensure their personal security, and how to get support from their supervisor) helped build loyalty and affirmation that the program and government cared about their well being.

Social insights were also integrated into the recruitment of health workers, to ensure that they had a profile that each community felt would build trust: gender, ethnicity, religion, and age were considered on the basis social analysis and input from the community. In Pakistan, recruitment of >8000 community-based vaccinators, >65% of whom are women, in addition to the communication network that was already in place (COMNet), have been critical to improving trust and reducing poliovirus in the past 2 years.

In Pakistan, once the recruitment and training strategy of health workers began to be realized in the field, a mass media campaign was overhauled with a new brand that promoted health workers as *sehat muhafiz* (“protectors of health”) and showed them as integral parts of the community (mothers, fathers, and daughters themselves), gender appropriate, ethnically relevant (>90% of cases occurring in 2013 and 2014 were among Pashtuns), and demonstrating genuine concern for children while vaccinating them. This cohesive communication approach that leveraged (1) mass media to create pride in the act of vaccination, (2) interpersonal communication to establish trust at the doorstep, and (3) community engagement to strengthen social norms in targeted areas is the first time such an integrated approach has been implemented with such precision to overcome chronic barriers to OPV vaccination.

Appealing to social expectations and professional standards can lead to significant improvements in the actions of providers. When providers act in the best interests of their patients, their patients are likely to notice and increase their trust in the advice provided by these same providers, which should lead to further improvements in health outcomes.

Whereas increasing information or knowledge is often not helpful or sufficient, simply reminding health workers of social expectations about their performance can improve it [7].

Pakistan has 56% fewer polio cases in 2016 than it did at the same time in 2015 [15]. In a 2016 poll, 72% of caregivers in the districts at highest risk for polio said their experience with vaccinators was “better” than their experience with those who visited their homes a year earlier (ie, before the strategy was rolled out) [16].

The revamped communication strategy has yet to be rolled out cohesively in other polio-affected countries. Hopefully, immunization and other emergency health programs can leverage the benefits of an approach that uses a strategic framework and multiple communication channels to prompt positive change at all social levels and that leads to greater success during each health worker–caregiver interaction.

## CONCLUSION

The polio eradication program has consistently demonstrated the same principle over 28 years: in institutions and communities alike, it is people, supported or constrained by the contexts in which they can make decisions, which determines success or failure. Health and immunization programs seeking to invest in a more effective and sustainable model should prioritize behavioral and social factors from the inception of program design. The polio experience demonstrates that programs where social evidence is integrated from the outset can be more effective, particularly in the most complex cultural and social contexts. This lesson should inform further efforts to strengthen routine immunization and broader health interventions [17]. Initiatives to mainstream human factors into program design should be well resourced and held to the same level of professional standards expected from more-traditional aspects of health programs [18]. Programs seeking to invest in a more robust evidence base for vaccine demand or outbreak response should not only build systems that can collect rigorous, reliable social data but must simultaneously invest in health systems and organizational cultures that are held accountable for implementing health programs that incorporate epidemiological and social data. These integrated health programs should enforce independent quality checks on how all available data have been interpreted and used and should develop planning cycles that allow for iterative adjustments based on new data. To succeed, this new model must remove the financial and reputational disincentives to learn from failure; they must be equipped to make timely adjustments if data come back demonstrating that an intervention or assumption requires modification.

To truly place human factors at the forefront of health and immunization programs, we must be willing to listen to what the people we are trying to serve actually want, even when this defies our own expertise and our individual experience. Recent global public health emergencies (eg, Ebola and Zika) have reiterated the need to put human, social, and behavioral dynamics at the center of a health response [19].

## Notes

***Supplement sponsorship.*** This work is part of a supplement coordinated by the Task Force for Global Health with funding provided by The Bill and Melinda Gates Foundation and the Centers for Disease Control and Prevention.

***Potential conflicts of interest.*** The husband of G. S. has done consulting work for Eli Lilly. All other authors report no potential conflicts. All authors have submitted the ICMJE Form for Disclosure of Potential Conflicts of Interest. Conflicts that the editors consider relevant to the content of the manuscript have been disclosed.
